# Impact- and Thermal-Resistant Epoxy Resin Toughened with Acacia Honey

**DOI:** 10.3390/polym15102261

**Published:** 2023-05-10

**Authors:** Ivana Stajcic, Filip Veljkovic, Milos Petrovic, Suzana Veličkovic, Vesna Radojevic, Branislav Vlahović, Aleksandar Stajcic

**Affiliations:** 1 Department of Physical Chemistry, “Vinča” Institute of Nuclear Sciences—National Institute of the Republic of Serbia, University of Belgrade, Mike Petrovića Alasa 12-14, P.O. Box 522, 11001 Belgrade, Serbia; filipveljkovic@vin.bg.ac.rs (F.V.); vsuzana@vin.bg.ac.rs (S.V.); 2 Faculty of Technology and Metallurgy, University of Belgrade, 11000 Belgrade, Serbia; mpetrovic@tmf.bg.ac.rs (M.P.); vesnar@tmf.bg.ac.rs (V.R.); 3 Mathematics and Physics Department, North Carolina Central University, Durham, NC 27707, USA; vlahovic@nccu.edu; 4 Center for Microelectronic Technologies, Institute of Chemistry, Technology and Metallurgy—National Institute of the Republic of Serbia, University of Belgrade, 11000 Belgrade, Serbia; stajcic@nanosys.ihtm.bg.ac.rs

**Keywords:** bio-modifier, acacia honey, epoxy resin, thermal stability, impact-resistant

## Abstract

High performance polymers with bio-based modifiers are promising materials in terms of applications and environmental impact. In this work, raw acacia honey was used as a bio-modifier for epoxy resin, as a rich source of functional groups. The addition of honey resulted in the formation of highly stable structures that were observed in scanning electron microscopy images as separate phases at the fracture surface, which were involved in the toughening of the resin. Structural changes were investigated, revealing the formation of a new aldehyde carbonyl group. Thermal analysis confirmed the formation of products that were stable up to 600 °C, with a glass transition temperature of 228 °C. An energy-controlled impact test was performed to compare the absorbed impact energy of bio-modified epoxy containing different amounts of honey with unmodified epoxy resin. The results showed that bio-modified epoxy resin with 3 wt% of acacia honey could withstand several impacts with full recovery, while unmodified epoxy resin broke at first impact. The absorbed energy at first impact was 2.5 times higher for bio-modified epoxy resin than it was for unmodified epoxy resin. In this manner, by using simple preparation and a raw material that is abundant in nature, a novel epoxy with high thermal and impact resistance was obtained, opening a path for further research in this field.

## 1. Introduction

Epoxy resins are widely used materials, due to their suitable properties such as a high Young’s modulus, chemical and thermal resistance, good corrosion resistance, and excellent insulation [1,2,3,4,5,6]. They were extensively studied for various industrial applications, including construction, automobile manufacture, and military and aerospace industry applications, which require high-performance materials [7,8,9]. In order to satisfy the application demands, numerous epoxy matrix composite materials with a versatility of ceramic and organic nanoparticles and fiber reinforcements were developed over the years [10,11]. However, cross-linked epoxy shows low toughness, which raises the concern of the composites’ performance under impact. The formation of micro-cracks that are not visible to the naked eye leads to a sudden breakage, which poses a great problem for manufacturers of parts that are placed in critical mechanical points. The heterogeneous nature of microscale epoxy-based composites could also be regarded as a weakness. Fiber-reinforced composites show high impact resistance in the direction of fiber orientation, while in other directions, damage occurs at low impact. As crack initiation and propagation take place in various ways, such as epoxy cracking, fiber failure, or debonding, it is very difficult to build a prediction model for fracture behavior and the potential absorbed energy [12]. The common challenges with conventional reinforcements include deformation and oxidation of polymer-based fibers, depending on the working conditions. Moisture acts as a plasticizer in epoxy resin, which leads to lowering the glass transition temperature value (T_g_) and reducing thermal and mechanical performance. Moisture can also induce corrosion in fiberglass, a frequently used reinforcement.

Recently, much attention was given to chemical modifications of epoxy in order to increase its thermal and mechanical properties. Meanwhile, growing environmental concerns, along with finite petrochemical resources, led to a focus on the development of composite materials with reinforcements from renewable resources [13]. In order for such materials to reach their full potential for application, a balance between mechanical performance and environmentally friendly behavior must be achieved. Flax or bamboo fibers, as well as bio-based silica, were proposed as green reinforcements to improve epoxy’s mechanical properties [14,15,16]. It was proven that the effect of various modifying additives and fillers (of natural origin) on the physicochemical and mechanical properties of epoxy polymer composites is determined by many factors. Bekeshev et al. modified diorite with phenyl-terminated agents in order to improve filler/polymer interface interaction, which resulted in increased thermal resistance and mechanical strength [17]. Oladele et al. incorporated eggshell and sisal fibers in an epoxy matrix, improving tensile and flexural strength [18]. The addition of bael shell biochar, proposed by Minugu et al., significantly increased the tensile strength and thermal stability of epoxy [19]. Bezerra et al. recently discovered that the high-load (30 wt%) addition of arapaima fish scales caused structural and mechanical changes in epoxy, resulting in increased hardness [20].

Lignin is one of the main plant constituents, with a high content of functional groups that make it suitable for use as a raw material in the synthesis of bio-based epoxy, as a replacement for the diglycidyl ether of bisphenol A (DGEBA) [21]. However, lignin shows low reactivity, resulting in solid products with low solubility [22]. Lu et al. functionalized epoxy in order to obtain a liquid, which is a more compatible epoxidized compound for the use as a bio-based epoxy. Recently, Xu et al. used gallic and modified tannic acid to synthesize bio-based epoxy resin with increased toughness [23]. Hollande et al. synthesized renewable epoxy bisphenols from ferulic acid and fatty acids [24]. They showed that thermal properties and degradability could be controlled by the different structure and content of the constituents. Although these resins have great potential, the glass transition temperature (T_g_) is very low, reducing the temperature range for application. Yu et al. prepared bio-based dimer acid diglycidyl ester for the toughening of DGEBA-based epoxy resin [25]. With a small percentage of the toughener, the impact strength was increased by more than two times. However, the T_g_ decreased, compared to the T_g_ with the unmodified epoxy. A similar trend was observed in soybean oil and saturated-fatty-acids-based modifiers prepared by Hu et al., except that the overall T_g_ values were significantly higher when compared to those in a previous study [26]. In order to achieve high-impact strength and compatibility DGEBA-based epoxy resin, Zhang et al. synthesized xylitol-based hyperbranched epoxy toughener, increasing the impact strength by over 200% [27]. With the same aim, Yadav et al. developed bio-rubber from soybean oil, increasing the critical strain energy by 500% [28]. Furthermore, Mi et al. modified konjac fly powder and achieved and increase in impact strength of over 100% with 20% of the toughener in DGEBA [29]. They attributed the improved impact behavior to the free-volume increase that resulted in internal stress reduction. Based on these studies, it can be seen that bio-based modifications of epoxy represent a great challenge with great potential for application and for the development of green technologies.

Honey is known for heterogeneous structure, with reactive components that could be used in materials chemistry. Along with carbohydrates such as fructose and glucose, many other substances can be found in honey, depending on the plant source and geographical origin [30].

Along with that, honey is a low cost, low moisture containing and abundant in nature raw material [31]. In the light of these facts, acacia honey was used in this research as a modifier for DGEBA based epoxy, with the aim of improving poor impact resistance. Acacia honey was chosen as a monofloral honey, common in Serbia. It was added in DGEBA/DETA system during the cross-linking at room temperature, and the resulting material was investigated from the thermal, mechanical, and structural aspect. After preparing samples with 1, 3, and 6 wt%, epoxy modified with 3 wt% of honey was chosen for further investigations that revealed high thermal and mechanical performance, opening the path for further research in this direction.

## 2. Materials and Methods

### 2.1. Materials

Diglycidyl ether of bisphenol A (DGEBA)-based epoxy resin and diethylenetriamine (DETA)-based hardener were purchased from R&G Faserverbundwerkstoffe GmbH, Composite Technology, Waldenbuch, Germany. Acacia honey, manufactured by ‘Mepolis’, Belgrade, Serbia (glucose + fructose: >70%, water: <20%, sucrose < 5%, 1.36 g/cm^3^) was used as the epoxy modifying agent.

### 2.2. Preparation of Samples

**Scheme 1:**
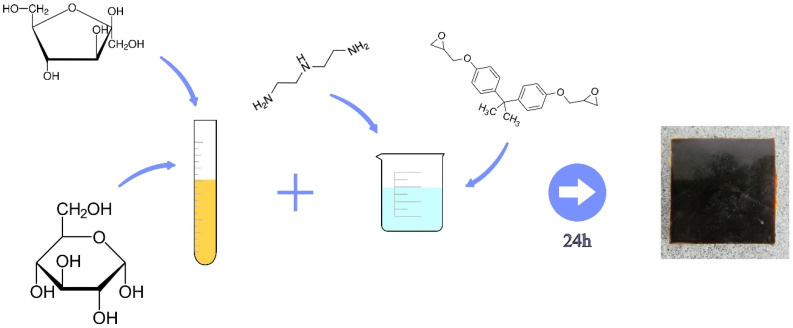
Preparation of bio-modified epoxy.

First, DGEBA and DETA were mixed in 20:9 volume ratios, in accordance with manufacturer’s instructions. After 2 minutes of vigorous mixing, 1, 3, or 6 wt% of acacia honey was added, and the mixing was continued for another 2 minutes. The mixture was poured into a silicone mold and left for 24 h at 25 °C to cross-link and solidify. Compared to the unmodified epoxy, gel time was reduced from 40 to 15 minutes. The samples were prepared for a controlled energy impact test with dimensions 60 mm × 60 mm × 6 mm. Scheme 1 illustrates the preparation of modified epoxy. Unmodified epoxy was labeled as E; epoxy modified with acacia honey was labeled in accordance with honey concentration: EH1 for 1 wt%, EH3 for 3 wt%, and EH6 for 6 wt% of honey.

### 2.3. Characterization of E and EH

Morphological differences between E and EH were recorded with a Field Emission Scanning Electron Microscope—FESEM (TESCAN MIRA 3, Brno, Czech Republic), surfaces were sputtered with gold. Structural changes with the addition of honey to epoxy resin were investigated using Fourier transform infrared—FTIR (Hartmann & Braun, MB-series, Bockenheim, Germany), 4000 and 400 cm^−1^, resolution of 4 cm^−1^. The thermal stability of EH was monitored using a Simultaneous Thermal Analyzer (STA) (TGA-DTG-DTA) (Netzsch STA 449 Jupiter F5, Bayern, Germany), in the temperature range from room temperature to 600 °C, with a heat rate of 10 °C/min. The sample mass was 10 ± 0.5 mg. The measurements were performed in synthetic air (21 vol% O_2_ + 79 vol% N_2_).

The difference in impact resistance of E, EH1, EH3, and EH6 was investigated with the High Speed Puncture Impact Testing Machine (HYDROSHOT HITS-P10, Shimadzu, Kyoto, Japan). Absorbed energy (total absorbed energy-E_tot_ and energy at maximum load-E_fmax_) values were calculated automatically from the load-time diagram. The diameter of the striker was 12.7 mm, with a hemispherical head; the set impact force was 10 kN. For samples E and EH1, impact velocity and depth were 0.05 m/s and 1 mm, respectively. A variation of impact depth and velocity (Table 1) was performed for samples EH3 and EH6, in order to investigate impact resistance.

## 3. Results and Discussion

### 3.1. FESEM Analysis

**Figure 1:**
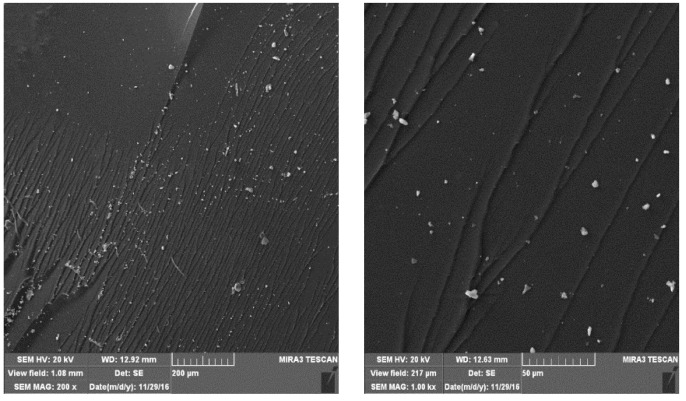
Fracture surface of E.

**Figure 2:**
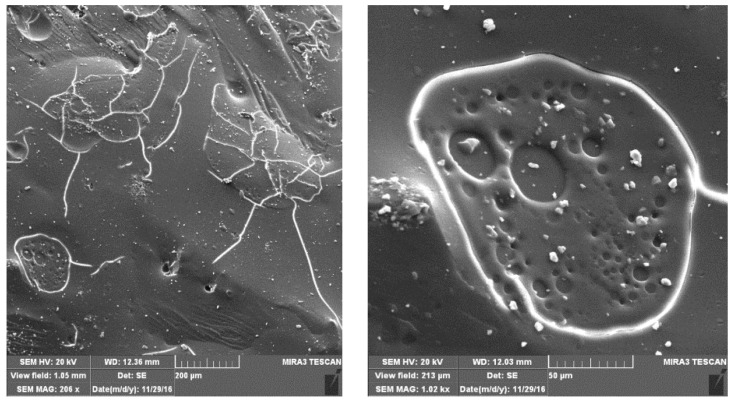
Fracture surface of EH3.

The toughening effect of honey addition to epoxy could be analyzed through the crack propagation pattern observed on FESEM images (Figure 1 and Figure 2). Depending on the crack path, there are different toughening mechanisms. In our case, the fracture surface of E shows typical brittle failure with polymer plastic deformation causing river-like path lines (Figure 1). This type of crack spreading is correlated with low fracture toughness [32].

On the other side, the fracture surface of EH3 showed higher roughness, which was directly connected to an increased toughness in the material. The surface was covered with cracks with a changeable path, with separate phases acting as tougheners. Obviously, crack arrest occurred around the phase-separated microstructural formations. The secondary phase was not separated from the rest of the surface during impact, indicating high compatibility between epoxy and the modified moiety. The dominant toughening mechanism was crack-branching that caused local energy dissipation and the formation of secondary cracks. In this manner, epoxy modified with acacia honey showed a crack propagation pattern similar to the nanocomposites with incorporated rigid nanoparticles [33,34]. In addition, the strong plastic deformation of EH3 also indicated higher toughness compared to E.

**Figure 3:**
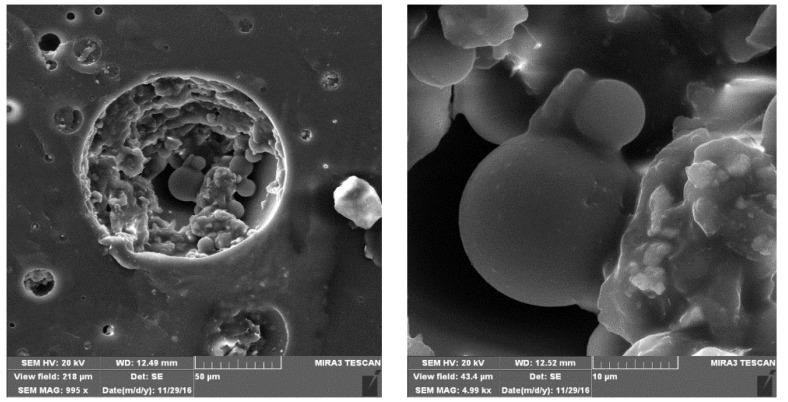
FESEM images of microcapsules at the surface of EH3.

On the part of the upper surface of EH3, pores filled with phase-separated spheres were found, presumably of the partly unreacted honey, which formed microcapsules (Figure 3).

Sample EH6 (Figure S1) showed the presence of large pores filled with partly unreacted honey throughout the entire volume, which was expected to result in lower mechanical performance compared to EH3.

### 3.2. FTIR Analysis

**Figure 4:**
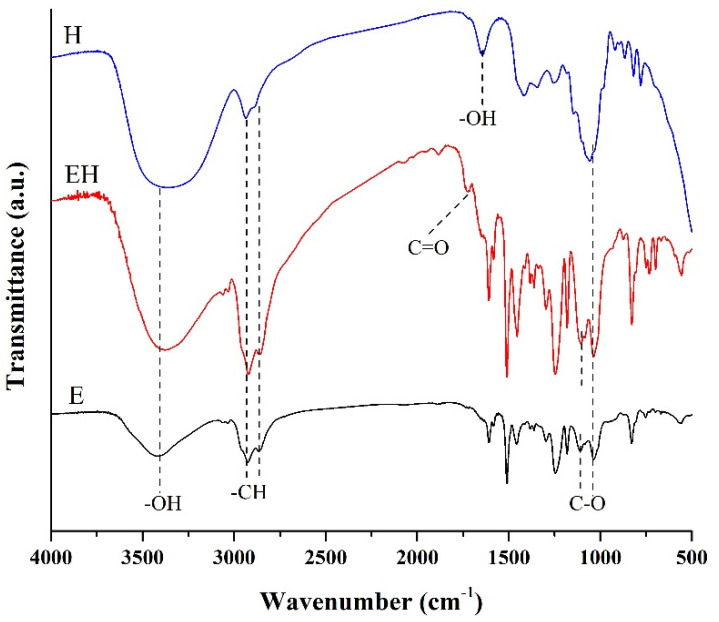
FTIR spectra of pure epoxy, honey, and bio-modified epoxy.

Honey is mostly composed of water and simple sugars, such as fructose and glucose, although it contains proteins, vitamins, minerals, and many other substances in small amounts [31,35]. The heterogeneity of acacia honey structure enabled different sets of complex chemical reactions when it reached contact with a suitable medium. In our case, we presumed that after DGEBA-based epoxide was mixed with DETA based cross-linker, the exothermic reaction of cross-linking was enough to initiate Maillard reaction of glucose and fructose in honey with –NH_2_, resulting in the formation of aldol condensation products [36]. Scheme 1 shows the sample of modified epoxy, where the brown coloration characteristic of Maillard reaction products can be observed [37]. In order to establish structural changes in epoxy after the addition of honey (H), FTIR spectroscopy was performed (Figure 4). The spectrum range (4000 to 400 cm^−1^) was divided into two regions to highlight structural differences between E and EH.

#### 3.2.1. The Region 4000–1300 cm^−1^

All spectra have broad bands from the H bonded –OH group in the region from 3350 to 3400 cm^−1^. However, in the spectrum of AH, this band was broader, indicating a higher concentration of hydroxyl groups that may originate from present moisture and carbohydrates. Aromatic –CH stretch was present in E and EH at 3063 cm^−1^ and 3032 cm^−1^, coming from phenolic groups in epoxy. Asymmetric –CH stretching can be seen in all the spectra around 2935 cm^−1^, as well as symmetric at 2870 cm^−1^.

E and EH3 showed small, broad bands around 1950 cm^−1^ and 1884 cm^−1^, originating from double-bonded carbon atoms. The most interesting structural change was observed by the appearance of a peak at 1721 cm^−1^, which comes from carbonyl groups that formed after the reaction of H and E. This peak was assumed to be from aldehyde C=O, which formed after the reaction of reducing sugars with amine [38]. In the spectrum of H at 1646 cm^−1^, a peak appeared that is related to the presence of moisture in H, which could overlap with 1° amide C=O, presumably from the proteins [39,40].

#### 3.2.2. The Region 1300–400 cm^−1^

In the region from At 1245 cm^−1^, 1184 cm^−1^, 1107 cm^−1^ and 1033 cm^−1^, C–O stretching vibrations were observed in the spectra of E and EH3 [41,42,43]. The H sample had only two peaks in that range, at 1252 cm^−1^ and 1052 cm^−1^, C–O stretching from C–OH, characteristic for fructose and glucose carbohydrates. The absence of a band around 1185 cm^−1^ proved that our H sample did not contain sucrose [39]. Out-of-plane –CH bending was observed in all three spectra in the region 916–697 cm^−1^ [44,45,46]. Stronger peaks in EH3 indicate the presence of carbohydrates [47].

### 3.3. Thermogravimetric Analysis (TGA)

**Figure 5:**
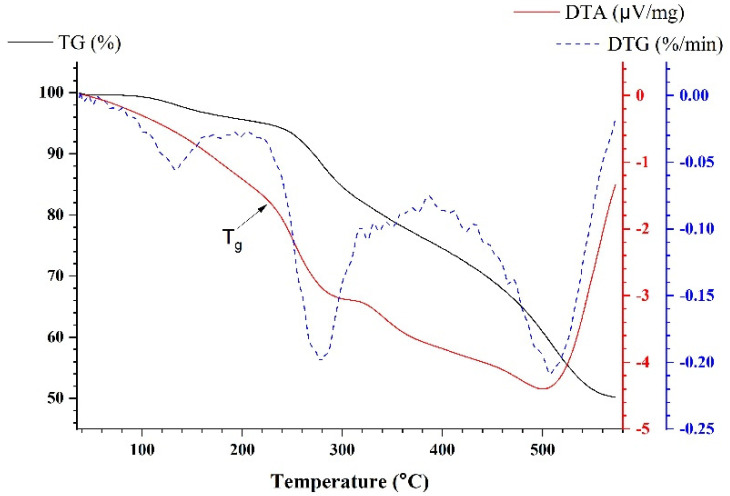
TGA-DTG-DTA curves for EH3.

In order to investigate the thermal stability of epoxy modified using acacia honey, simultaneous thermal analysis was performed in the air. Following the assumptions made by structural and morphological analyses, structures formed during processing should have resulted in bio-modified epoxy resin with high thermal stability. The curves obtained from TGA-DTG-DTA for the most representative sample, EH3, are presented in Figure 5. The TGA curve of EH3 showed a weight loss of 2.7% in the region around 35–165 °C, which indicates a very low presence of water and unreacted compounds with a low boiling point [48]. A small DTG peak was detected at 133 °C in this region. The degradation of epoxy moiety due to thermal oxidation reactions that involve chain scission was observed in two stages [45]. The first stage was from 165 °C to 390 °C, with a strong DTG peak at 279 °C, which is in accordance with the literature and our previous investigation on epoxy thermal stability [49,50]. Weight loss in this region was 22%, which corresponds to a minor endothermic peak at DTA.

The second degradation stage was from 390 to 570 °C, where the largest weight loss of 25% occurred, with a strong DTG peak at 508 °C [51]. In this region, the degradation of epoxy benzene rings occurred [52].

The char yield at 570 °C was 50%, which was significantly higher compared to the previous research regarding modified epoxies with improved thermal stability [25,53]. This stability could be explained by highly stable condensed structure that evolved during the initial heating of the sample [54,55]. This stability is similar to the reported phenolic condensed structures, which indicates that reactions including the DGEBA moiety resulted in highly stable modified epoxy [56]. T_5_, T_10_, and T_30_ are presented in Table 2.

The heat resistance index (T_hri_) (Table 2) was calculated according to Equation (1) [52]:

(1) $$T_{hri}=0.49\;\times \;[T_{5}+0.6\times \left(T_{30}-T_{5}\right)]$$

This value was comparable to epoxy reinforced with multi-walled carbon nanotubes, which revealed the potential of honey as a modifier. Compared to the highly stable DGEBA epoxy used by Mi et al., T_5_ and T_10_ of EH3 were lower, but compared to their reported T_max_ value of 368 °C, EH3 T_50_ of 570 °C was substantially higher [29].

The glass transition temperature (T_g_) determined by DTA (Table 2) was significantly higher compared to the commercial epoxy, especially since T_g_ depends on a curing temperature, which was 25 °C in our case [57,58,59].

For comparison, values for neat epoxy presented in Table 2 (Supplementary Material, Figure S2) show that T_5_, T_10_, and T_30,_ and T_hri_ were much lower than EH3. T_50_ was 382.7 °C. With the exception of T_50_, values were quite low compared to the cured DGEBA resin found in the literature, probably due to less cross-linking achieved without higher-temperature post-curing [29,60]. The same was observed for the neat epoxy T_g_ value (Figure S3, Table 2), which was more than 20 °C lower compared to the epoxy used by Yu et al. [25].

### 3.4. Impact Test

The energy absorbed during the impact can be divided in the following phases:

- The first phase represents the energy absorbed through the initial creation of the crack;
- The second phase represents the energy absorbed during the propagation and development of the crack until the failure of the material.

The first phase involves the elastic response of the material and plastic deformation, which is characteristically low for fully cured epoxy resins. The maximum load (F_max_) is reached at the end of this phase. The energy absorbed by material at this moment is denoted as E_fmax_. The second phase starts when the crack forms, leading to a significant deterioration of mechanical properties until the breakage or rupture. Total absorbed energy (E_tot_) represents energy absorbed from the beginning of the controlled energy impact test until the load drops below zero, which is considered the end of the test. There are four common types of failure, corresponding to the material’s plastic deformation propensity. The first one is brittle failure, typical for ceramic materials and rigid polymer structures, such as cross-linked polymers that form 3D covalently bonded networks. This type of failure is described by no or very small plastic deformation with fast fracture, and low E_tot_ value. Brittle-ductile fracture is the second type, characterized by a small plastic deformation before breakage. Materials that show ductile-brittle failure (third type) have the ability to plastically deform and absorb more energy during the impact. Extensive plastic deformations are distinctive feature of the fourth type of failure, corresponding to the large E_tot_ value.

**Figure 6:**
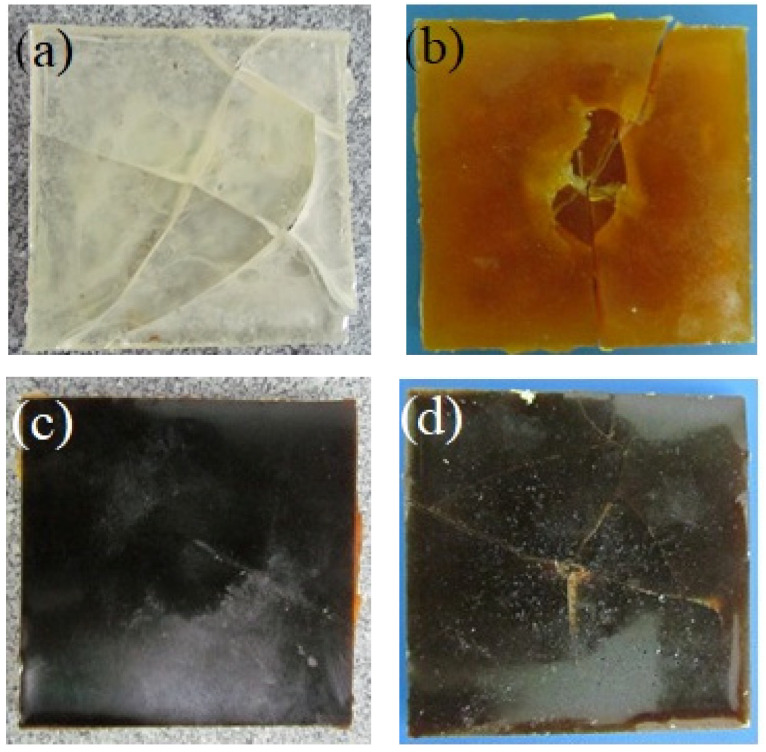
Photographs of: (**a**) E, (**b**) EH1, (**c**) EH3, and (**d**) EH6 after the first impact.

**Figure 7:**
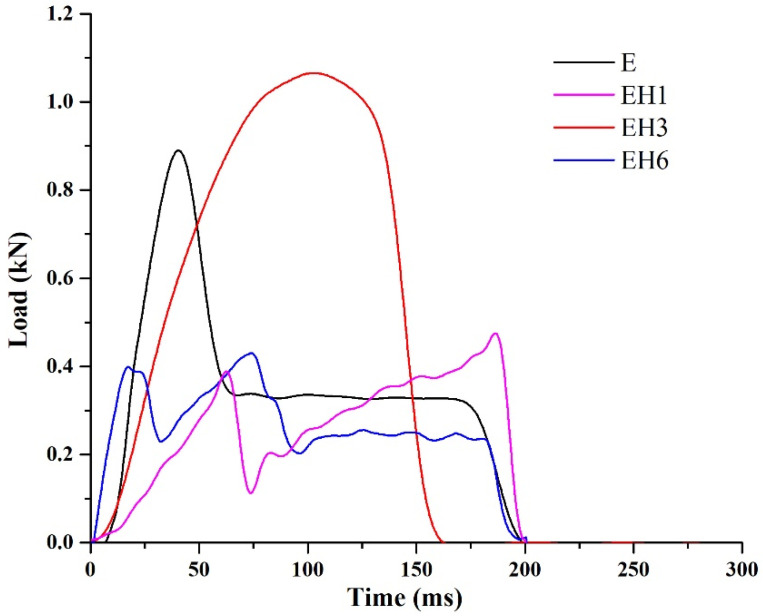
Load-time curves from the first impact by the controlled energy impact test.

Figure 6 shows photographs of E, EH1, EH3, and EH6 after the first impact, while corresponding load-time curves obtained from the controlled energy impact test can be seen in Figure 7. The surface of EH6 (Figure 6d) was covered with discontinuous pores as a consequence of increased phase separation in bio-modified resin. Unmodified epoxy showed typical ductile failure because full cross-linking could not be achieved without high-temperature post-curing [40]. After the first impact, samples E and EH1 were completely destroyed. On the other hand, it can be seen that EH3 was barely damaged after the first impact, while EH6 was damaged but not completely destroyed. Furthermore, EH3 showed more brittle-ductile failure, which is in accordance with thermal analysis that indicated the existence of a rigid condensed structure, compared to the ductile failure of room-temperature cured E, which showed poor thermal stability. This occurrence lead to performing series of impacts in order to establish the impact resistance potential of EH3.

**Figure 8:**
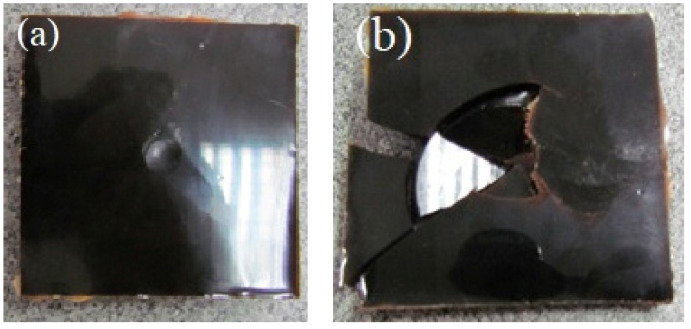
Photographs of EH3: (**a**) after the 7th impact and (**b**) punctured EH.

EH3 was punctured eight times with different impact velocities and puncture depths. After the seventh impact, lower absorbed energies were measured as a consequence of the initiated damage from the previous impacts. The sample was left at room temperature for 24 h, after which EH3 returned to its original shape. On the other side, EH6 failed after the second impact, which was consistent with the indications made after the morphological analysis. Figure 8 shows photographs of EH3 after the seventh impact and punctured EH3.

**Figure 9:**
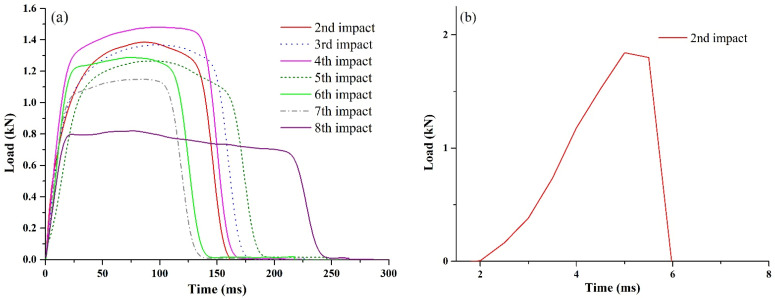
Load-time curves from controlled energy impact test for (**a**) EH3 and (**b**) EH6.

**Figure 10:**
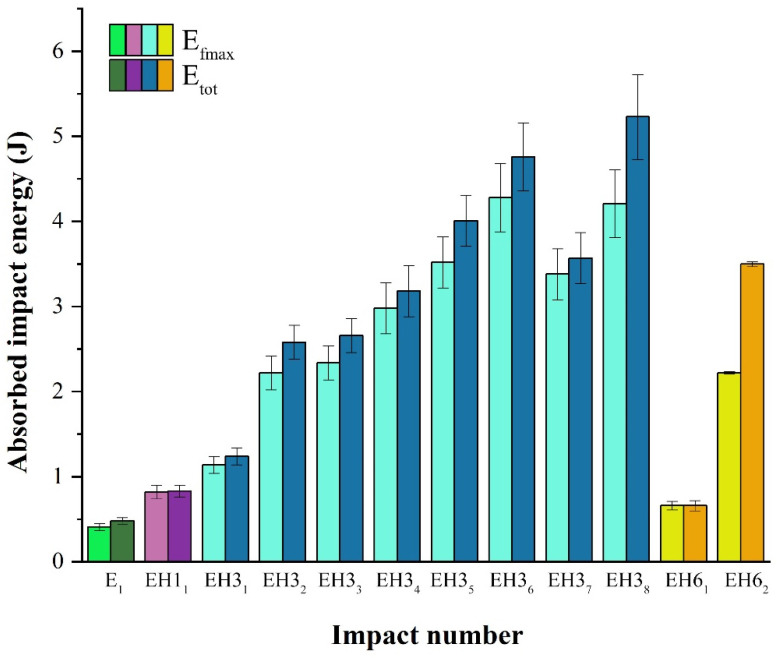
Absorbed energy values for E, EH1, EH3, and EH6.

Figure 9 represents load-time curves from the second to eighth impact of EH3 (Figure 9a) and the second impact of EH6 (Figure 9b). E_fmax_ and E_tot_ for all the samples are presented in Figure 10. During the first impact, E absorbed 0.48 J with the failure of the material, which is consistent with the low toughness of neat epoxy observed on FESEM images [32]. EH1 showed a transition from ductile to brittle failure, with approximately two times higher absorbed energy than E, while EH3 absorbed around 2.5 times more energy with barely visible damage. EH6 showed weaker toughness compared to EH1 and EH3, absorbing 0.66 J. However, unlike EH1, it was not completely broken after the first impact.

On second impact, both E_fmax_ and E_tot_ increased in EH3, to 2.22 J and 2.58 J, respectively. The brittle-ductile failure mode remained, in the absence of material failure. The shape of the load-time curve varied from impact to impact, from brittle-ductile to ductile. At the seventh impact, both absorbed energy values decreased compared to the sixth impact, which indicates the beginning of EH3 failure. E_fmax_ decreased by 20%, E_tot_ by 25%, but the sample did not break. However, after the sample was left for 24 h at room temperature, impact strength recovery occurred, which was demonstrated by the highest E_fmax_ and E_tot_ values observed at the final, eighth impact, where the 1 m/s impact velocity was set and the depth was 6 mm, leading to a puncture of EH3.

EH6 showed brittle failure during the second impact, as a consequence of the large crack that formed after the first impact. The absorbed energy increased to 3.50 J, but the sample was destroyed, which could be a consequence of an increased phase separation caused by unreacted components. This separation leads the a formation of large pores that could have weakened the sample after the crack initiation (Figure S3). According to the impact test results, 3 wt% is the optimal concentration of honey in epoxy for achieving highly impact-resistant bio-modified resin.

## 4. Conclusions

This study investigated the potential of honey as a toughener for DGEBA-based epoxy resin. After the addition of different amounts of honey to the DGEBA/DETA system, aldol condensation products formed, which were observed as a separate phase that was responsible for the toughening of the resin. Structural analysis revealed the formation of an aldehyde carbonyl group at 1721 cm^−1^, which presumably formed after the reaction of sugars in honey with DETA. Thermal analysis of the sample with 3 wt% of honey showed a high char yield of 50% at 570 °C, which is a consequence of DGEBA moiety participation in the reaction with honey, resulting in stable phenolic condensed structures. The heat resistance index was 173.8 °C, which is considered very high, as was the glass transition temperature of 228 °C. The influence of honey concentration in epoxy on impact resistance was investigated with a controlled energy impact test, and it was established that bio-modified epoxy with 3 wt% withstood several impacts without failure, absorbing the highest amount of energy during the final stroke, leading to the conclusion that it was the optimal amount for obtaining significantly toughened epoxy resin. In light of the presented results, honey proved to be an excellent bio-modifier, which also contributes to the expansion of investigation in the field of bio-based tougheners for polymeric materials.

## Figures and Tables

**Table 1:** Impact test parameters.

Sample	EH3		EH6	
Impact	Impact Velocity, m/s	Impact Depth, mm	Impact Velocity, m/s	Impact Depth, mm
1	0.05	1	0.05	1
2	0.1	2	1	6
3	0.2	2	-	-
4	0.2	3	-	-
5	0.3	3	-	-
6	0.5	3	-	-
7	0.5	6	-	-
8	1.0	6	-	-

**Table 2:** Comparison of thermal analysis data for EH3 and E.

	T_5_, °C	T_10_, °C	T_30_, °C	T_hri_, °C	T_g_, °C
EH3	221.5	271.1	443.5	173.8	228.0
E	115.3	244.7	290.3	107.9	102.8

## Data Availability

The data presented in this study are available on request from the corresponding author. The data are not publicly available due to privacy.

## Supplementary Materials

The following supporting information can be downloaded at: https://www.mdpi.com/article/10.3390/polym15102261/s1, Figure S1: FESEM image of EH6; Figure S2: TGA curve for neat epoxy in air; Figure S3: DSC curve for neat epoxy in air.
