# Supplementary material for: Impact- and Thermal-Resistant Epoxy Resin Toughened with Acacia Honey
Source: Polymers (Basel). 2023 May 10;15(10):2261. doi: 10.3390/polym15102261 (PMC10222091; doi:10.3390/polym15102261)
Supplement: Supplementary file 1 [file polymers-15-02261-s001.zip › polymers-2360844-supplementary.pdf]

Impact- and Thermal-Resistant Epoxy Resin Toughened with Acacia Honey

**Ivana Stajcic [1,*], Filip Veljkovic [1], Milos Petrovic [2], Suzana Veličkovic [1], Vesna Radojevic [2], Branislav Vlahović [3]
and Aleksandar Stajcic [4]**

[1] Department of Physical Chemistry, "Vinča" Institute of Nuclear Sciences—National Institute of the Republic of Serbia, University of Belgrade, Mike Petrovića Alasa 12-14, P.O. Box 522, 11001 Belgrade, Serbia;
filipveljkovic@vin.bg.ac.rs (F.V.); vsuzana@vin.bg.ac.rs (S.V.)
[2] Faculty of Technology and Metallurgy, University of Belgrade, 11000 Belgrade, Serbia; mpetrovic@tmf.bg.ac.rs (M.P.); vesnar@tmf.bg.ac.rs (V.R.)
[3] Mathematics and Physics Department, North Carolina Central University, Durham, NC 27707, USA; vlahovic@nccu.edu
[4] Center for Microelectronic Technologies, Institute of Chemistry, Technology and Metallurgy—National
Institute of the Republic of Serbia, University of Belgrade, 11000 Belgrade, Serbia; stajcic@nanosys.ihtm.bg.ac.rs
* Correspondence: ivana_r@vinca.rs

S3. Results and discussion

S3.1. FESEM analysis

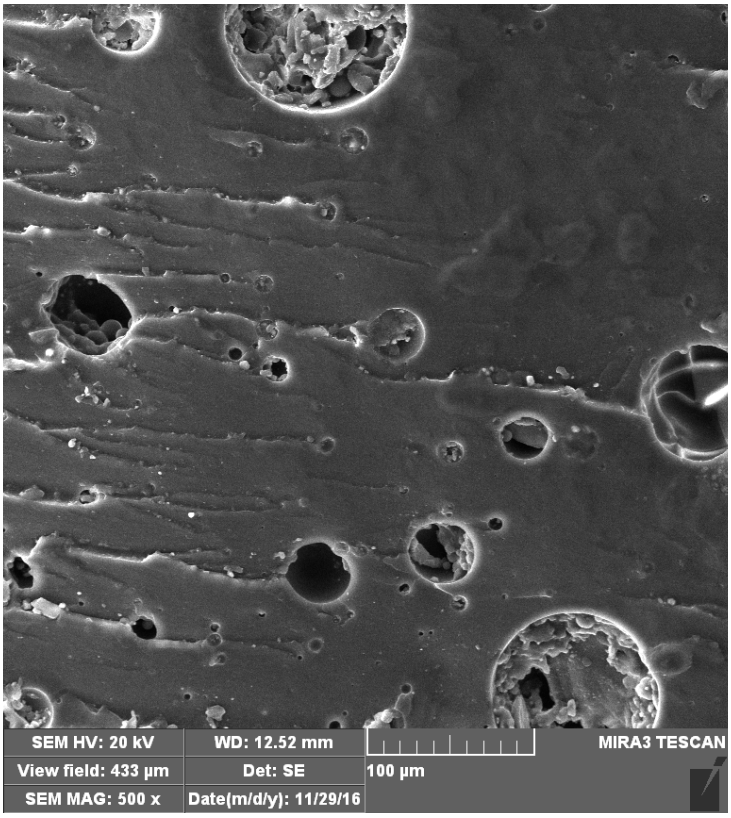

Figure S1. FESEM image of EH6.

S3.2. Thermogravimetric analysis (TGA) and differential scanning calorimetry (DSC)

Thermal analysis of epoxy was performed on a device for thermogravimetric analysis (TGA) in the temperature range from 24 ˚C to 500 ˚C (SDT Q600, TA instruments), under air, with heat rate set at 10 ˚C/min. DSC analysis was performed on a TA Instruments TA DSC Q10 calibrated with indium. The temperature range was from 30 °C to 250 °C with a heating rate of 10 °C/min and air flow of 50 mL/min. The samples were heated to 250 °C, held at 250 °C for 5 min to cancel the temperature history, and then cooled to 30 °C at the same cooling rate. Reheating to 250 °C was then carried out. The transformation temperature was determined as the midpoint of the initial transition slope of the curve.

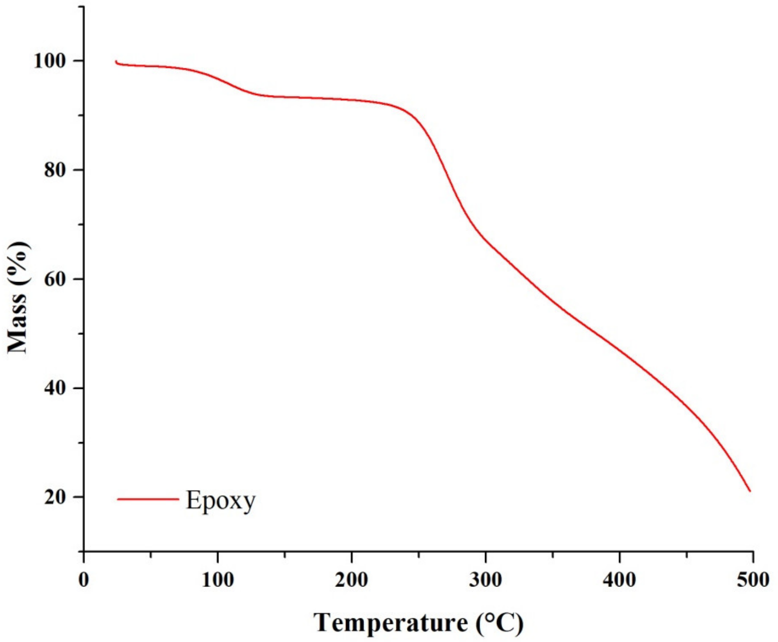

Figure S2. TGA curve for neat epoxy in air.

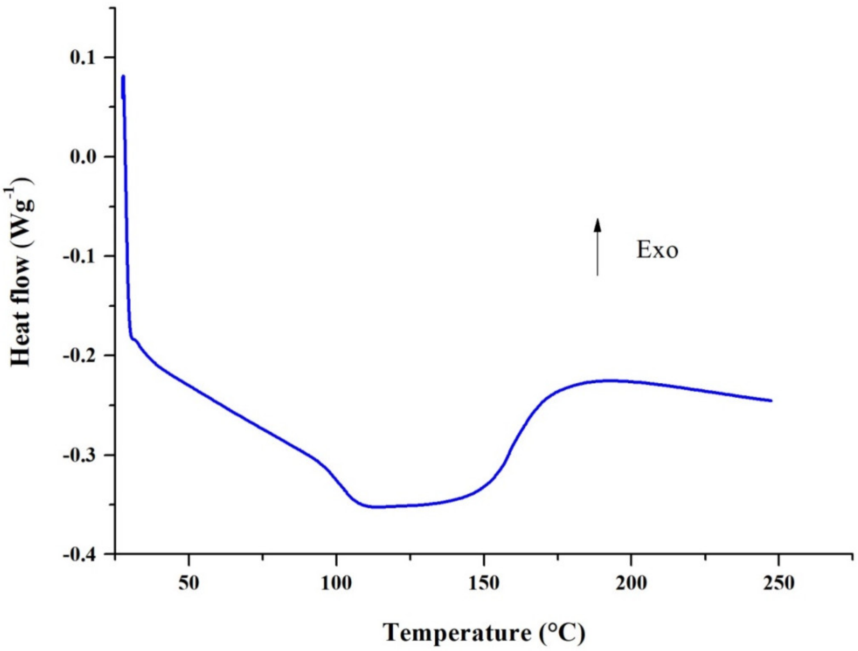

Figure S3. DSC curve for neat epoxy in air.
